# A Novel Promising Endometrial Preparation Protocol for Frozen-Thawed Embryo Transfer: A Randomized Controlled Trial

**DOI:** 10.3389/fendo.2021.730059

**Published:** 2021-09-20

**Authors:** Jian-Chun Li, Yan-Hong Wang, li-Ying Peng, Yun Zhou, Shi-Bin Chao

**Affiliations:** ^1^Department of Clinical Laboratory, The First Affiliated Hospital of NanChang University, NanChang, China; ^2^ART Center, Maternal and Child Health Care Hospital, ShangRao, China; ^3^Department of Reproductive Medicine, Reproductive Hospital Affiliated to Jiangxi University of Traditional Chinese Medicine, Nanchang, China; ^4^Department of Obstetrics and Gynecology, Fuzhou Medical College of NanChang University, Fuzhou, China

**Keywords:** frozen-thawed embryo transfer, endometrial preparation, endometrial receptivity, embryo implantation, *in vitro* fertilization, down regulation ovulation induction

## Abstract

**Background:**

In recent years frozen-thawed embryo transfer (FET) has played an increasingly important role in ART, but there is limited consensus on the most effective method of endometrial preparation (EP) for FET. Inspired by significantly higher implantation rate and clinical pregnancy rate of the depot GnRH-a protocol, we proposed a novel EP protocol named down-regulation ovulation-induction (DROI) aimed to improve pregnancy outcomes of FET.

**Methods:**

This was a single-center, randomized controlled pilot trial. A total of 307 patients with freeze-all strategy scheduled for first FET were enrolled in the study. A total 261 embryos were transferred in DROI-FET group including 156 patients and 266 embryos were transferred in mNC-FET group including 151 patients. Reproductive outcomes were compared between the two groups.

**Results:**

The basic characteristics of patients, and the average number, quality and stage of embryos transferred were comparable between the two groups. Our primary outcome, implantation rate(IR) in DROI-FET group, was significantly higher than that of the mNC-FET group (54.41% versus 35.71%, P<0.01). The clinical pregnancy rate (CPR) and ongoing pregnancy rate (OPR) in DROI-FET group was also higher than that in mNC-FET group (69.87% versus 50.33%, P<0.01; 64.10% versus 42.38%, P<0.01).

**Conclusion(s):**

Compared to existing endometrial preparation methods, the DROI protocol might be the more efficient and promising protocol.

## Introduction

With the application of vitrification techniques and widespread ongoing adoption of freeze-all strategy, the number of FET cycles has dramatically increased (1). Whether in fresh ET or in FET, embryo implantation relies upon embryo quality, endometrial receptivity, and synchronization between embryo and endometrium (2). From the beginning of IVF, embryo quality has been the most emphasized factor (3), while more and more studies are paying attention to endometrial receptivity (4). But there is no consensus on either the explicit mechanism or the solution to improve endometrial receptivity up to now (5, 6).

FET cycle overcomes some adverse effects of fresh ET, such as reducing the risk of ovarian hyperstimulation syndrome (OHSS) and avoiding some negative effects on the endometrium. FET resulted in higher clinical pregnancy rate than did fresh ET (2, 7). This has fuelled the call for a new strategy called freeze-all strategy where no fresh embryo transfer is conducted and all available embryos are cryopreserved, and transferred in subsequent FET cycles (2, 8).

FET protocols are relatively simple in the attempt to prepare the endometrium (9). Various endometrial preparation regimens have been developed for FET. The most commonly applied protocols include natural cycle FET (NC-FET), modified NC-FET using HCG, hormone replacement therapy (HRT) cycle with or without GnRH agonists, and ovulation induction cycle. Nevertheless, by far, there is no superiority of any regimen for EP over another one in terms of reproductive outcomes (9, 10).

Recently, several studies reported that depot GnRH-a COS protocol yields relatively high CPR and IR in fresh ET (5, 11, 12). Lei J reported that IR per fresh ET cycle were 51% in the depot GnRH-a protocol, 37% in the GnRH antagonist, and 45% in the long GnRH-a protocols, respectively (P<0.01) (12). The higher IR is considered to be through the way of improving endometrial receptivity, because the outcomes of the embryos derived from the COS cycles might not be superior to those from GnRH antagonist cycles in subsequent FET cycles (11, 13, 14). The latest research published in June 2020 elucidated the mechanism by which depot GnRH-a protocol improves endometrial receptivity. The protocol enhanced several well-established biomarkers for endometrial receptivity such as HOXA10, MEIS1 and LIF gene expression and thereby helps to improve endometrial receptivity (12).

Based on the suggestion that depot GnRH-a protocol might improve endometrial receptivity, we proposed a novel EP protocol named down-regulation ovulation -induction (DROI). The purpose of the present study was to testify whether the novel protocol could improve IR, CPR and OPR compared to modified nature cycle (mNC) EP protocol.

## Materials and Methods

### Study Design and Participants

This is a single-centre RCT performed at the ART centre of ShangRao Maternal and Child Health Care Hospital, China. The minimal sample size calculated with a significance level of 0·05 and with statistical power of 80% is 973 for each group (15). Ideally, sufficient sample size would have been recruited to this research to determine equivalence between the two EP protocols. So this study can thus only be viewed as a pilot RCT study.

The study conformed to the ‘Declaration of Helsinki for Medical Research involving Human Subjects’ and was approved by the hospital’s ethics committee. Infertile women undergoing first FET cycles in the period from March 2018 to May 2020 were enrolled. This RCT trial was registered at the Chinese Clinical Trial Registry, number ChiCTR2000039804. The candidate patients obtained detailed information of both protocols, including the duration of the down-regulation and the potential risk of pituitary suppression. All patients enrolled gave written informed consent for the procedures.

A total of 360 patients with freeze-all strategy scheduled for first FET were randomly assigned to two study groups in a 1:1 ratio. Participants in the RCT Random allocation was performed by a study doctor at endometrial preparation by means of computer generated random numbers in sealed, unlabelled envelopes. Doctors, patients and nurses administering the interventions were not blinded to the treatment assigned. Inclusion criteria were age<38 years, normal menstrual cycle, BMI 18–28 kg/m2, and basal FSH level<10 IU/ml. Patients diagnosed with poor ovarian response (antral follicle count (AFC) <7 follicles or anti-Müllerian hormone (AMH) <1.1 ng/ml), hyperprolactinemia, endometriosis, hydrosalpinx and uterine abnormalities, thyroid disease were excluded.

### COS and Embryo Vitrification, Thawing, and Transfer

All participants were given GnRH antagonist regimen or Progestin-primed ovarian stimulation (PPOS) for ovarian stimulation as extensively described elsewhere (2, 16). When at least two follicles were 18 mm or greater in mean diameter, human chorionic gonadotropin (HCG) at a dose of 4000–10000 IU or 0.2 mg of triptorelin was administered to induce the final maturation of oocytes. Oocyte retrieval was performed 34–36 h after trigger. Embryo morphology was assessed and graded on Day 3 according to the Cummins criteria (17). Generally, two Grade I or Grade II embryos were vitrified on Day 3, and supernumerary embryos were cultured continuously until the blastocyst stage on Day 5 or Day 6 before vitrification. The vitrification procedure was performed following standard protocols using Kitazato Freeze Kit (Kitazato Corporation, Janpan). Embryos containing ≥7 cells, with ≤20% fragmentation and symmetric or slightly asymmetric blastomere distribution, but without multinucleation on Day 3 and blastocysts graded ≧ 4BB according to Gardner morphological criteria were classified as top-quality embryos (2).

### Endometrial Preparation Before Embryo Transfer

**Figure 1:**
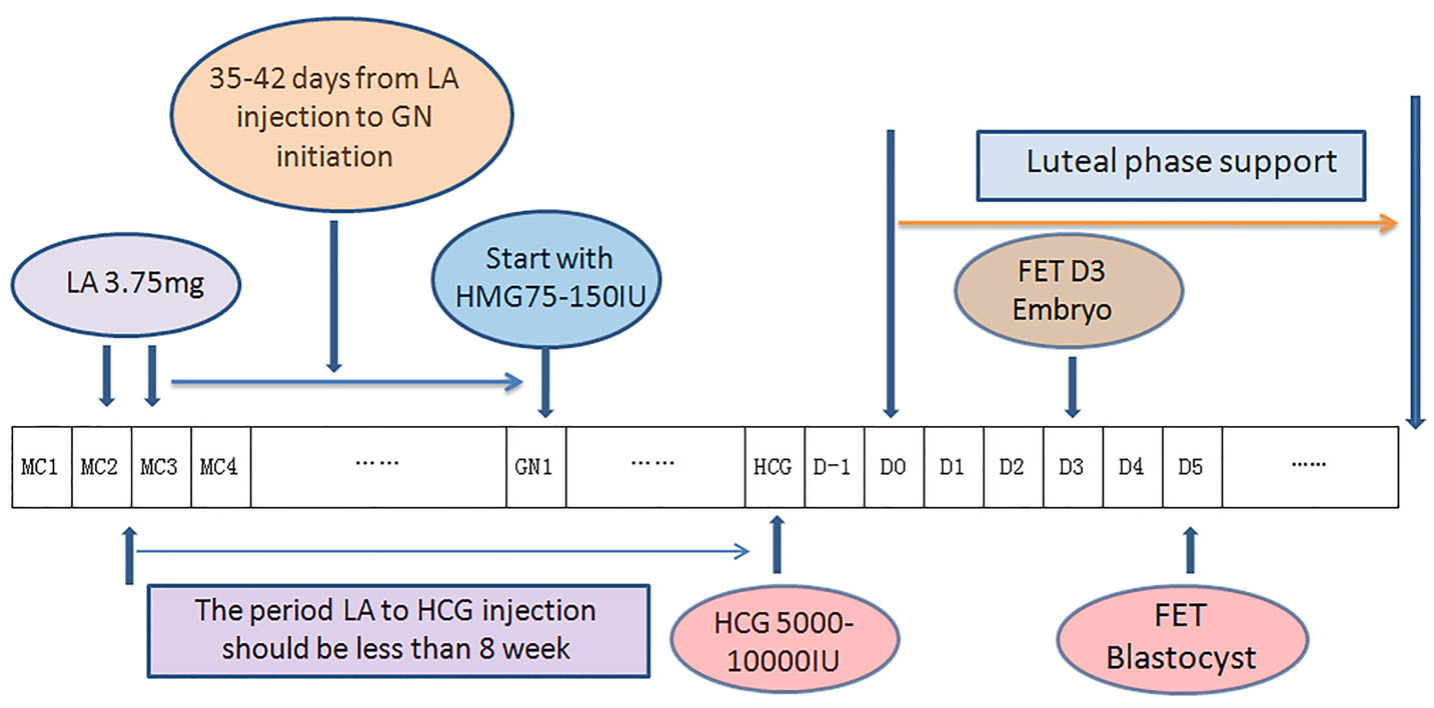
Flow chart of down-regulation ovulation-induction procedure. One depot of 3.75 mg leuprorelin acetate was injected at the 2nd or 3rd day of the menstrual cycle, in which ovarian stimulation with 75–150 IU Gns would start 35–42 days later along with confirmation of pituitary downregulation. Gonadotropin stimulation continued until endometrial thickness ≥7 mm. A dose of HCG 5000-1000 IU was injected at 9:00 p.m. and ET was arranged 5 days later for D3 embryos or 7 days later for blastocysts. Progesterone supplement was initiated 2 days after ovulatory trigger as fresh ET. (LA, leuprorelin acetate; GN, Gonadotropin; HCG,Human Chorionic Gonadotropin; MC, Mnstrual Cycle; D0, theoretical oocyte retrieval day).

In the DROI-FET group, pituitary down-regulation was achieved by full dose (3.75 mg) of Leuprorelin Acetate (Lizhu Pharmaceutical Trading Co, Shanghai, China) at day 2 or day 3 of the menstrual cycle. Gonadotropin stimulation started after 35-42 days when the biggest antral follicle was larger than 5-6 mm. Patients received 75–150 IU of HMG injection (Lizhu Pharmaceutical Trading Co, ZhuHai, China) daily according to AMH, AFC, especially ovarian response in the COS cycles. The specific starting GN dose was that when ≥ 10 oocytes were harvested in the fresh cycle, the starting dose was about half of the COS starting dose, and when < 10 oocytes, the starting dose was about two-thirds of the COS starting dose. Gonadotropin stimulation was adjusted according to transvaginal ultrasound and serum E2, LH and P levels. Gonadotropin stimulation continued until endometrial thickness ≥7 mm and meeting one of the following two criteria: (1) If there were leading follicles, the number of these leading follicles which had a mean diameter of ≥16 mm was between 1-3, with serum estradiol level 200-800ng/l and progesterone level <1.5ng/mL; (2) If there was no dominant follicle ≥16 mm, at most four follicles reached the diameter between 12 and 15mm, with serum estradiol levels between 150 and 1000ng/l and progesterone level <1.5ng/mL. A dose of HCG 5000-10000 IU (Lizhu Pharmaceutical Trading Co.) was injected at 9:00 p.m. and ET was arranged 5 days later for D3 embryos or 7 days later for blastocysts. Progesterone supplement was initiated 2 days after ovulatory trigger as fresh ET described elsewhere (6). Procedures of DROI-FET protocol is summarized in **Figure 1**.

Patients undergoing modified NC-FET (mNC-FET) attended for ultrasound evaluation between days 10 and 12 of their menstrual cycle to confirm follicular growth and endometrial thickness. Participants commenced self-monitoring of ovulation by using urinary dipsticks. Ultrasound monitoring continued until the dominant follicle reached 16–18 mm and endometrial thickness ≥7 mm. A dose of HCG 5000-10000 IU (Lizhu Pharmaceutical Trading Co) was given subcutaneously to trigger ovulation. In both DROI-FET and mNC-FET groups, luteal support was continued to 10 weeks of gestation if a pregnancy occurred.

### Outcome Parameters and Statistical Analysis

The primary outcome of the study was implantation rate. The secondary end points included clinical pregnancy rate and ongoing pregnancy rate.

Continuous data were compared with the Student t test and categoric variables were compared with the χ2 test. Multivariable logistic regression analysis was used to evaluate the possible relationship between the protocol of endometrial preparation and CPR, OPR after adjusting for confounding factors, including age, BMI, BFSH, infertility duration, endometrium thickness, number of embryos transferred, and embryo developmental stage. All statistical analyses were performed by using the Statistical Package for Social Sciences (SPSS) version 22.0. A P value < 0.05 was considered to be statistically significant.

## Results

### Study Population

**Figure 2:**
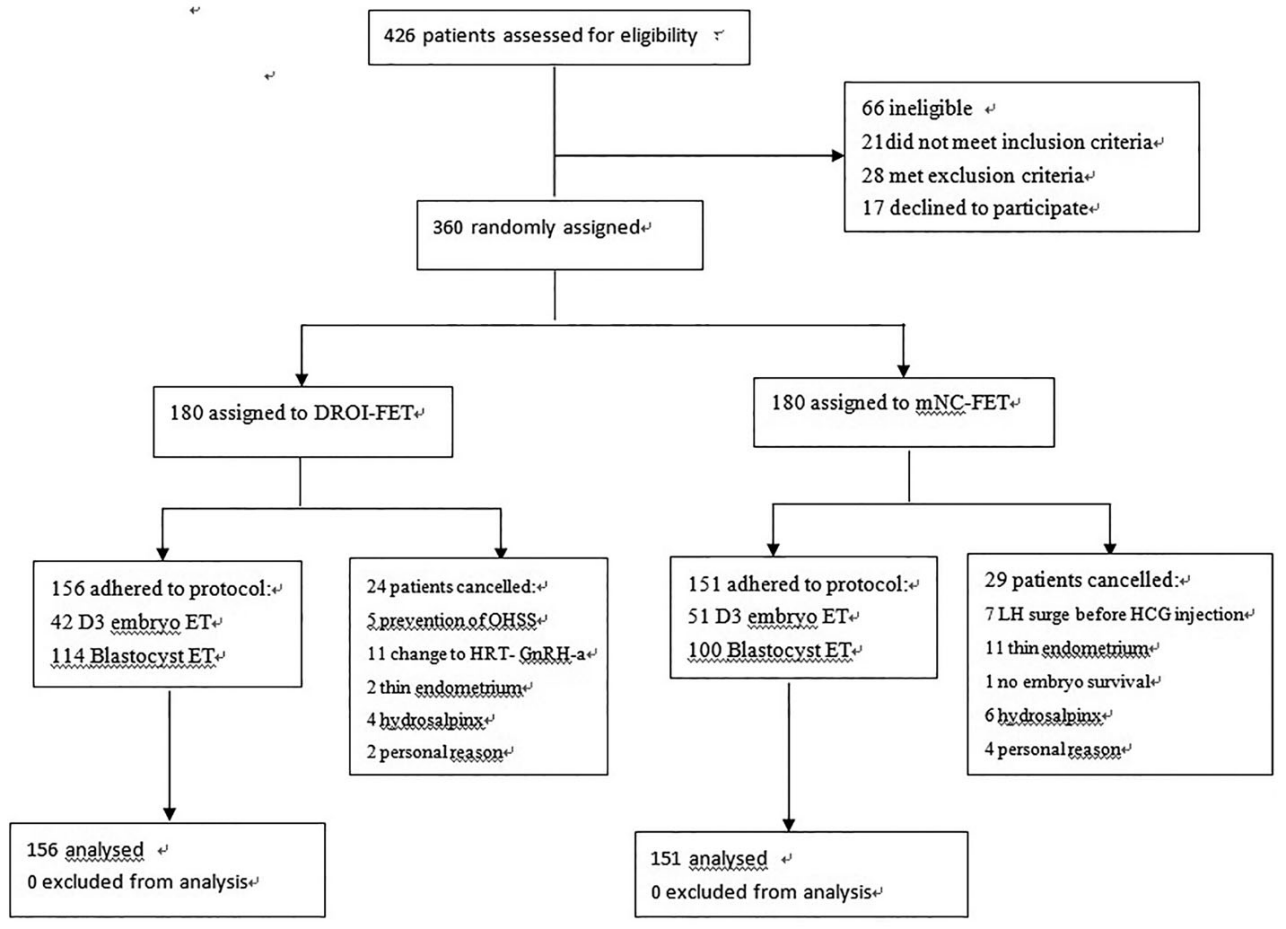
Flowchart showing of enrollment and randomization of study patients.

A total of 360 women were randomized and 307 had ET and completed the study. Reasons for dropout are summarized in **Figure 2**. Remaining patients received treatment according to study group allocation, resulting in 156 patients (50.81%) receiving DROI-FET and 151 (49.19%) receiving mNC-FET.

### Baseline Characteristics

Patients’ baseline characteristics are detailed in **Table 1**. No significant difference was observed between the two treatment groups regarding age, BMI, duration of infertility, total antral follicle count, indication for IVF, or type of infertility. The baseline hormone profile and COS protocol in fresh cycles were similar between the two groups.

**Table 1:** Basic characteristics of patients at the cycle level.

Characteristic	DROI-FET (156)	mNC-FET (151)	P value
**Age (y)**	30.58 ± 4.43	30.50 ± 4.35	0.87
**Body mass index (kg/m2)**	21.27 ± 2.55	21.50 ± 2.35	0.41
**Infertility duration (years)**	3.33 ± 1.25	3.30 ± 1.43	0.84
**Type of infertility**			
**Primary**	72(46.15%)	71(47.02%)	
**Secondary**	84(53.85%)	80(52.98%)	0.88
**Indications for IVF**			
**Tubal factor**	101(64.74%)	83(54.97%)	
**Male factor**	30(19.23%)	28(18.54%)	
**Unexplained infertility**	6(3.84%)	5(3.31%)	
**Others**	19(12.18%)	35(23.18%%)	0.08
**Baseline sex hormone**			
**FSH (IU/L)**	6.57 ± 2.21	6.59 ± 2.09	0.94
**LH (IU/L)**	5.06 ± 2.21	5.19 ± 2.29	0.61
**E2 (pg/mL)**	32.95 ± 14.15	33.00 ± 15.27	0.98
**P (ng/mL)**	0.36 ± 0.25	0.34 ± 0.25	0.61
**Total antral follicle count**	12.83 ± 5.03	13.73 ± 4.34	0.94
**Protocol for COS**			
**GnRH-ANT**	101(64.74%)	86(56.95%)	
**PPOS**	55(35.26%)	65(53.05%)	0.16

Data are presented as mean ± SD for continuous variables and n (%) for dichotomous variables. All P values were assessed with the use of χ2 or Student t test. DROI, down-regulation ovulation-induction; mNC, modified nature cycle; FET, frozen-thawed embryo transfer.

### Cycle Characteristics of FET

The quantity, quality and developmental stage of embryos are the key factors that affect the outcome of FET. As presented in **Table 2**, the average number of embryos transferred in the DROI-FET group was a little less than that in the mNC-FET group, but there was no significance (1.67 ± 0.47 VS 1.76 ± 0.43, p > 0.05), and the number of double embryo transfers was higher in the mNC-FET group and neared significance with p=0.09. The proportions of patients with single embryo transfer, two-embryo transfer, transferred embryo stage at cleavage or blastocyst stage, and top-quality embryo transferred including cleavage stage and blastocyst stage between the two groups were comparable.

**Table 2:** Cycle characteristics at transfer level.

Characteristic	DROI-FET (156)	mNC-FET (151)	p value
**Average number of embryos transferred**	1.67 ± 0.47	1.76 ± 0.43	0.08
**Number of embryos transferred**			
**1**	51(32.69%)	36(23.84%)	0.09
**2**	105(67.31%)	115(76.16%)	
**TOP-quality embryo transferred**			0.08
**0**	7(4.49%)	10(6.62%)	
**1**	70(44.87%)	49(32.45%)	
**2 **	79(50.64%)	92(60.93%)	
**Embryo stage**			0.19
**Cleavage stage (day 3)**	42(36.84%)	51(33.77%)	
**Blastocyst (day 5)**	114(63.16%)	100(66.23%)	
**Endometrium Thickness on the day of HCG (mm)**	10.62 ± 1.43	10.51 ± 1.87	0.57

Data are presented as mean ± SD for continuous variables and n (%) for dichotomous variables. All P values were assessed with the use of χ2 or Student t test. Other abbreviations as in **Table 1**.

### Reproductive Outcomes of FET

Main reproductive outcomes of FET are presented in **Table 3**. Our primary outcome, implantation rate per embryo transferred in the DROI-FET group, was statistically higher than that in the mNC-FET group (54.41% versus 35.71%, P<0.01). The rate of clinical pregnancy and ongoing pregnancy in the DROI-FET group was also higher than that in the mNC-FET group (69.87% versus 50.33%,P<0.01; 64.10% versus 42.38%,P<0.01). 76 of 156 women in the DROI-FET group had singleton pregnancy, which was similar to that in the mNC-FET group (57 of 151, 37.75%). The rates of twin pregnancies were higher in DROI-FET group(21.15% versus 12.58%; p<0.05). There was 1 case of intrauterine and ectopic pregnancy in the mNC-FET group and this patient had been transferred two top-quality cleavage embryos.

**Table 3:** Reproductive Outcomes.

Characteristic	DROI-FET (156)	mNC-FET (151)	p value
**Implantation rate**	142/261 (54.41%)	95/266 (35.71%)	<0.01
**Clinical pregnancy rate**	109/156 (69.87%)	76/151 (50.33%)	<0.01
**Ongoing pregnancy rate**	99/156 (64.10%)	64/151 (42.38%)	<0.01
**Singletons**	76/156 (48.72%)	57/151 (37.75%)	0.05
**Twins**	33/156 (21.15%)	19/151 (12.58%)	<0.05
**Ectopic pregnancy**	0	0	
**intrauterine and ectopic pregnancy**	0	1	
**Moderate or severe OHSS**	0	0	

Data are presented as n (%) for dichotomous variables. All P values were assessed with the use of χ2. Other abbreviations as in **Table 1**.

After adjustment for the above-mentioned confounding factors (**Table 4**), the CPR remained consistently higher following DROI-FET (adjusted odds ratio [aOR] 2.73, 95% confidence interval [CI] 1.63-4.58). Furthermore, the OPR was significantly higher in DROI-FET compared with mNC-FET (aOR 2.59, 95% CI 1.58-4.26) after correcting for confounders.

**Table 4:** Unadjusted and adjusted odds ratios (ORs) of reproductive outcomes following DORI-FET versus mNC-FET.

Outcome	Unadjusted OR	Adjusted OR
	(95% CI)	(95% CI)
**Clinical pregnancy**	2.29	2.73
	(1.43-3.65)	(1.63-4.58)
**Ongoing pregnancy**	2.36	2.59
	(1.49-3.74)	(1.58-4.26)

Analyses were adjusted for age, body mass index, infertility duration, BMI,bFSH,endometrium thickness, number of embryos transferred, and embryo developmental stage. CI (confidence interval); Other abbreviations as in **Table 1**.

## Discussion

Since the first success of FET in 1983 (18), it has been widely used to increase the cumulative pregnancy rate without repeated oocyte retrieval procedure. FET contributes to elective single embryo transfer (eSET), avoiding multiple gestations (19), and it is a strategy to prevent OHSS or to cancel embryo transfer if the endometrial environment is not optimal for fresh ET (20). In recent years the number of FET cycles performed has increased dramatically due to the trend towards transferring fewer embryos after a fresh IVF cycle and freeze-all strategy widely accepted (10, 20). Endometrial preparation is the most critical step for FET. The options range from natural cycle FET, over ovarian stimulation, to HRT with GnRH or without GnRH pretreatment. Indeed, in the latest Cochrane review (9, 10), based on RCTs comparing different EP regimens for FET, it was concluded that no regimen was superior to another.

Among the three key factors that determine embryo implantation, endometrial receptivity is difficult to evaluate accurately because its mechanism is unclear and there are no objective evaluation criteria. The result of endometrial receptivity is embryo implantation, in return, embryo implantation rate reflects endometrial receptivity. Many studies and efforts have been made to improve endometrial receptivity but there is little consensus (21, 22). However, it has been reported that CPR and IR increase when patients are given a period of artificial amenorrhea caused by GnRH-a (23, 24). Depot GnRH-a protocol has been widely used in China and it dramatically increases the CPR and IR in fresh ET (5, 6, 12). The success of the protocol is thought to be due to improved endometrial receptivity instead of embryo quality because the embryos derived from the protocol have no advantage over those from other COS protocols in FET cycles (25–27). Therefore, we proposed the DROI EP protocol derived from the depot GnRH-a COS protocol to take advantage of the favorable endometrial receptivity.

As expected, the data in the present study showed that IR in the DROI-FET group increased significantly compared with the mNC-FET group. The novel EP method (DROI) mimics the depot GnRH-a procedure but provides a more favorable endocrine environment instead of the supra-physiological level which might lead to endometrium gene expression and structural abnormalities (28).

The only risk of the DROI protocol may be a possibility of OHSS. It should be kept in mind that the DROI was proposed as an EP protocol for FET. So the principle is that as long as the endometrial thickness is up to 7mm, estrogen is up to 150-200pg/l, HCG could be injected to prevent risk of OHSS. In our study, there was no case with moderate or severe OHSS in both groups.

The strength of this study is that we propose a novel and promising endometrial preparation protocol completely different from existing EP protocols. However, there are limitations in this study. First, it is a small sample size, single-centre pilot study which may be subject to selection bias. Second, miscarriage rate and live birth rate are not yet presented in the study. Larger sample size and a more detailed study are very much needed to verify the practicability of the protocol in future studies.

## Conclusion

Inspired by the depot GnRH-a COS protocol, we proposed a novel endometrial preparation protocol named DROI for FET. Moreover, our RCT data showed that the DROI increased CPR, OPR and especially IR dramatically compared to the mNC. The effects might be associated with improving endometrial receptivity. Given the fact that there is little consensus on the best protocol for existing EP protocol, the DROI might be the more efficient and promising protocol for FET.

## Data Availability Statement

The raw data supporting the conclusions of this article will be made available by the authors, without undue reservation.

## Ethics Statement

The studies involving human participants were reviewed and approved by maternal and child health care hospital’s ethics committee. The patients/participants provided their written informed consent to participate in this study.

## Author Contributions

Conceptualization: J-CL and S-BC. Data curation: Y-HW and YZ. Formal analysis: S-BC. Methodology: S-BC and Y-HW. Supervision: S-BC and Yun Zhou. Validation: S-BC and Y-HW. Writing – original draft: J-CL and L-YP. Writing – review & editing: S-BC. All authors contributed to the article and approved the submitted version.

## Funding

This study was supported by Key Research and Development projects of JiangXi Province, China (NO: 20171BBG70010, 20192BBG70005).

## Conflict of Interest

The authors declare that the research was conducted in the absence of any commercial or financial relationships that could be construed as a potential conflict of interest.

## Publisher’s Note

All claims expressed in this article are solely those of the authors and do not necessarily represent those of their affiliated organizations, or those of the publisher, the editors and the reviewers. Any product that may be evaluated in this article, or claim that may be made by its manufacturer, is not guaranteed or endorsed by the publisher.
